# Specific Learning Disorders and Special Educational Needs during COVID-19 Pandemic; Pilot Survey Study Performed in Local District Schools in Italy

**DOI:** 10.3390/children9060825

**Published:** 2022-06-02

**Authors:** Flavia Marchese, Assunta Grillo, Massimo Pettoello Mantovani, Giulia Gargano, Pierpaolo Limone, Flavia Indrio

**Affiliations:** 1 Department of Medical and Surgical Science, Pediatric Section, University of Foggia, Viale Pinto 1, 71122 Foggia, Italy; flavia.marchese@hotmail.it (F.M.); assuntagrillo88@gmail.com (A.G.); mpm@unifg.it (M.P.M.); 2 European Pediatric Association, Union of National European Pediatric Societies and Associations, 10115 Berlin, Germany; 3 Association pour l’Activité et la Recherche Scìentifiques, 2067 Nouchatel, Switzerland; 4 Department of Surgical and Medical Science Foggia, University of Foggia, 71122 Foggia, Italy; 5 Italian Society of Pediatrics, 00042 Rome, Italy; 6 Italian Academy of Pediatrics, 20121 Milan, Italy; 7 Learning Science Institute, University of Foggia, Via Arpi, 71122 Foggia, Italy; giulia_gargano.563473@unifg.it (G.G.); pierpaolo.limone@unifg.it (P.L.)

**Keywords:** specific learning disorders, special education needs, COVID-19, dyslexia, dysgraphia, dyscalculia

## Abstract

Specific Learning Disorders (SLD) are a group of heterogeneous health deficits frequently diagnosed in early childhood that cause difficulties in general intellectual functioning. In the last decades in Italy new laws have been developed to give practical guidelines for the best education plans for children with SLD. Background: The aim of our study was to determine the efficacy of the educational treatment on SLD in Primary and Secondary schools in the Italian city of Barletta. We acquired valuable data to evaluate Special Education Needs during COVID-19. Methods: Our study was conducted from April to June 2021, during the second “lockdown” period in Italy. A fact-finding survey was conducted to schools with a questionnaire provided to the teachers to acquire data on the SEN applied in the management of distance learning for children. Results: The study involved 15 male and 6 female pupils with SLD in Primary Schools and 18 male and 6 female in Secondary Schools. The schools participating in the study organized distance learning programs with a support teacher with a 1:1 ratio. Data showed that all children with SLD needed a support teacher. Conclusions: The findings of this pilot study suggest that distance learning programs are able to achieve adequate educational goals, despite the difficulties of the lockdown period.

## 1. Introduction

Specific Learning Disorders (SLD) are a set of heterogeneous health deficits that may impair the ability to read, write, calculate, listen, and use verbal expression. They include dyslexia, dysgraphia, dyscalculia, and dyspraxia. The term specific means that the disorder only affects some abilities, leaving intact the general intellectual functioning. Moreover, they are evolutionary, because they vary with age of the subject. A child may have trouble in understanding instructions, carrying out tasks, and in interactions with others.

A child may have to do more efforts in memorizing information, talking to others and understanding what others are saying to them. Consequently, they may have very low self-esteem or show symptoms of anxiety [1].

Italian Law no. 170/2010 provides the right to education in the school to students with SLD. The Italian Minister of Education Decree no. 5669/2011 gives practical guidelines on what needs have to be guaranteed to students with SLD [2]. Thanks to these laws, students with SLD can be supported by Special Educational Needs (SEN) programs.

Special education is therefore a training provision which is extra or different from what is needed by other children the same age. Some children may need sign language, worksheets in a larger font, or one-to-one/small group support [2]. 

Children with SEN often have learning difficulties. Learning difficulties are classified in three grades: moderate if a child may take longer to learn skills than the majority of their peers; severe if a child has significant learning impairments and is likely to require high levels of specialist support in school; and profound and multiple learning difficulty when children have complex learning needs, physical difficulties, or severe medical conditions. 

In Italy there is an inclusive school system, in which students with and without SEN learn together. Students with SEN are supported by special education teachers, who prepare different educational plans for their own students [2].

In different European countries, such as Germany, students with SEN attend a special school through the decision of the parents or legal guardians [3].

There are different support focuses for students with SEN: learning, emotional, and social development; speech, sight, hearing; mental, physical, and motor development and instruction for sick students [3,4]. Data from the literature show that technical skills of schools are positively enhanced if significant attention, feedback, and individual support for students is provided by teachers. To this regard, teachers plan and carry out specific activities that are required to achieve educational goals. To acquire academic skills, educational tools benefits are pursued by the use of practical or virtual materials (so-called manipulatives, e.g., blocks or play money). Moreover, in our opinion, in distance learning, the teacher and child with SEN perceive more difficulties in reading, writing, and mathematics due to a lack of use of concrete materials.

For students with SLD, the programs are to provide lessons and exams allowing additional time, oral rather than written tests, reading of texts in digital form, use of speech software, and the use of any other technology which was felt useful to facilitate the study and exams. Before the COVID-19 pandemic, in-person lessons supported students with SEN with the use of research tools and electronic resources available online, computers with speech programs, interlibrary lending services and audio books, and electronic copies of the articles [5]. 

One of the most socially relevant conditions of SLDs is dyslexia, as it is characterized by difficulties that impact on reading and writing abilities. The severity of dyslexia can vary from mild to severe and this learning disability persists throughout life. However, although diagnosed and supported by specialists, dyslexia is frequently not emphasized in class during the early grades of school, in order to avoid children becoming frustrated or being discriminated against by their school-mates due their possible difficulty in learning to read.

Our pilot study focused on SLD in Primary and Secondary schools in the Italian Southern city of Barletta. The aim was to evaluate whether the compensatory interventions provided to children in the pre-pandemic period and during the lockdown, as well as the educational treatment were able to ensure that effective preventive and protective measures were effective in helping children to better manage this condition, taking into account that psychological factors possibly caused by the pandemic may have influenced the results. The main objective was to acquire valuable data helpful to understand whether Special Education Needs programs, including distance learning, have been ensured during COVID-19. Finally, our survey investigated the possible negative impact and risks associated to distance teaching on SLD children. ADHD was not taken into account in the survey because COVID restriction legislation did not allow meetings in person.

## 2. Materials and Methods

### 2.1. Study Design and Sample

Our study was conducted from April to June 2021, during the second “lockdown” period in Italy. Out of the total 17 schools (8 Primary and 9 Secondary) active in the city of Barletta, 12 participated in the study (7 Secondary and 5 Primary). 

### 2.2. Survey Questionnaire

A fact-finding survey was conducted to schools. The questionnaire was designed in DanSurvey, a licensed product of Lime Survey 2.0 (Symfony, London, UK), which is a free and open-source online survey application written in PHP, based on a MySQL, PostgreSQL, or MSSQL database, and distributed under the GNU general public license [6]. The questionnaire was developed using web-based standard guidelines [7], used in previous studies published in literature [8]. The survey included questions about the total number of children in the schools, the number of children already diagnosed with SLD, the gender, the average age of onset of symptoms, the possible need for a support teacher, and the type of SEN that was guaranteed to them in the pre-pandemic period or during the lockdown. The questionnaire provided to the teachers was designed to acquire data on the SEN definition adopted in the different schools participating in the study in order to understand whether a unitary methodological approach was applied in the management of distance learning for children with SLD.

## 3. Results

The total number of children with SLD in Primary Schools was 21 out of a total of 726 subjects (2.8%), while in Secondary schools it was 24 children out of a total of 1160 (2.0%).

The study involved 15 male and 6 female pupils with SLD in Primary Schools and 18 male and 6 female students with SLD in Secondary Schools. The mean age at diagnosis was 7 years in Primary Schools and 8 years in Secondary Schools. 

We observed a difference rate in sex: 73% of these participants were male and 27% were female.

During COVID-19, the schools participating in the study organized distance learning programs with a support teacher with a 1:1 ratio. During the COVID-19 pandemic, a dedicated web platform was used for all children recruited in our studio. This teaching program was provided simultaneously with the rest of the classes. Data showed that all children with SLD needed a support teacher.

### Demographic Characteristics and Type of Disorders

Table 1 summarizes the data of the questionnaire related to related to the total number of children in the schools, the number of children diagnosed with SLD, the gender, and the average age of onset of symptoms observed in the 12 participating schools.

Some children had more than one deficit. A total of 27 students presented 2 symptoms, dyslexia and dysgraphia, and 3 had dyslexia and dyscalculia (Table 2).

## 4. Discussion

The results obtained in our study highlight the great impact that confinement has had on students with dyslexia and their families, but also the greater attention which Primary and secondary school examined in Barletta gave to children with SLD [9]. Our survey showed that all children who followed the dedicated platform achieved the expected results for their deficit, although they showed more difficulties in pursuing and achieving the results. As expected, children with dyslexia reported more difficulties in following online classes and managing homework compared to normally developing (so-called “healthy”) children. At the same time, however, children with dyslexia appeared to be less worried about the school closure: the school context implies the exposure to performance and social issues that usually trigger anxiety or feelings of inadequacy, therefore they may perceive the school closure as protective for them [10]. Our study showed that the role of distance learning has ensured the efficiency of learning school thanks to the technologies available, with which these children are familiar with, which included speech-to-text software that transforms homework reading in listening assignments, books or digital vocabularies, and concept maps.

Ahmed et al. defined COVID-19 as the most significant global health crisis of our generation [11]. For Shaw, it has subsequently had significant impacts on the mental wellbeing of healthcare practitioners [12].

During COVID-19 there was a need for a reorganization of SED to ensure safety measures and distancing. In March 2020, in full health emergency, the first operational indications for distance learning were provided, some possible actions were declined, the concept of working coherently with the reference context was reaffirmed, and teachers were invited to make effective choices from a didactic point of view and above all inclusively, with a strong emphasis on planning and teamwork. In particular, with regard to students with SLD, it is necessary to devote, in the planning and implementation of remote activities, particular attention to the presence in the classroom of students with SLD and to the respective personalized teaching plans [5,13].

The relationship between distance education and pupils with learning disabilities is complex and challenging. During the months of closure there were many solicitations for teachers, students, and their families to face the many problems that arose in itinere to make distance learning inclusive and effective. In fact, SLD students have more difficulties in mastering new and changing learning environments, but many of them have some strengths that also help them to thrive in this unprecedented learning environment. Teachers and parents who recognize these strengths can encourage students to translate these skills into the classroom in the future [14,15].

During COVID-19, SLD experts invited teachers to record lessons which could be always available to be listened to according to the times of each child, thus promoting inclusion and respecting the needs of each learner, especially those with SLD, since in distance learning the time and pace of online lessons was pressing and not adequate for the teacher–pupil relationship. In fact, as their educational needs require continuous dedicated and systematic care on a daily basis, children diagnosed with SLD may be at increased risk for detrimental learning consequences during the COVID-19 lockdown. The suspension of school in-person activities may had impacted the continuity of educational care for children with dyslexia with the risk of increased emotional and psychological burden related to social distances, lack of dedicated specialist support, and isolation. Parents of children with special educational needs exhibited worries about the possibility for their children to fall even further behind in school because they did not feel adequate they could meet their specific needs during the COVID-19 emergency. Additionally, worries about the lack of supervised and specialist care for their children’s disability condition and rehabilitation was the most significant predictor of parents’ stress, depressive, and anxious symptoms during the COVID-19 lockdown [14,16].

There have been a few perplexities in the post-pandemic period with the resumption of studies in-person—the return to previous organization of learning materials, and new reorganization of time [5].

The importance of diagnostic tests has been established subjected to children with SLD for the purposes of learning, but it is necessary to compare percentages of children with SLD between schools recruited in Barletta and those at a national level. Children with SLD in the participating schools were 5%, while at the national level, dating back to 2011, it was 4%. This difference, although not statistically significant, can be explained by the lack of knowledge of the condition studied. Finally, this study suggests that children suffering from LSD may have many opportunities to manage their condition thanks to the use of adequate compensatory tools, and thanks to diagnostic tests that enable families and caretakers to timely intervene in LSD children to properly manage their condition with the support of adequate supporting programs.

### Limitations of the Study and Directions for the Future Research

The authors are aware of the limitations of this study. This is a cognitive investigation with a small sample size, which limits the generalizability of the results. The timing of data recruitment met the pandemic period with the due restrictions of the DPCM, which influenced the participation of schools and prevented a direct interaction and testing of the children. However, the methodology used allowed the adequate collection of data through teachers. Authors should discuss the results and how they can be interpreted from the perspective of previous studies and of the working hypotheses. The findings and their implications should be discussed in the broadest context possible. Future research directions may also be highlighted.

## 5. Conclusions

Students with Special Educational Needs (SEN) are an especially fragile group, as they have significantly greater difficulties in learning than other groups of children and young people of the same age. During the COVID-19 pandemic, children had major learning difficulties rather than before lockdown, due to the need to use distance learning platforms provided by their schools.

Nonetheless, alternative forms of teaching, examination, and education dispensatory strategies were tailored to all students with SEN. There is, in general, a great need to identify the right tools and strategies to support students with difficulties in reading, writing, and mathematics, and provide them the right help to students with SEN. However, in our experience, thanks to the professional figure of the personal teacher who supported the children, they were able to reach the planned educational goals, despite the difficulties expected in pursuing their educational goals.

The findings of this pilot study suggest that distance learning programs are able to achieve adequate educational goals if suitable supporting strategies and programs are provided to children. This type of investigation should be expanded, and comparisons between schools of different areas encouraged. Further studies would enable the acquisition of valuable information to help develop recommendations to be adopted by educational institutions for students with SEN.

## Figures and Tables

**Table 1:** Total number of children of the schools, number of children diagnosed with SLD, gender, the average age of onset of symptoms.

5 Primary Schools	7 Secondary Schools
21 SLD/726 total	24 SLD/1160 total
Mean age at diagnosis: 7 years	Mean age at diagnosis: 8 years
15 SLD males/726 total	18 SLD males/1160 total
6 SLD females/726 total	6 SLD females/1160 total
21 SEN children	24 SEN children

SLD—Specific learning disorders; SEN—Special Education Needs.

**Table 2:** Number of children with specific learning disorders by type of disorder.

Type of Disorder	Number
Dyslexia	3
Dyscalculia	3
Dysgraphia	8
Dyslexia + Dysgraphia	18
Dyslexia + Dyscalculia	9

## Data Availability

Not available.
